# Intelligent metasurface realizes human robots with ‘brain’ and ‘limbs’

**DOI:** 10.1093/nsr/nwad064

**Published:** 2023-03-09

**Authors:** Xiangang Luo

**Affiliations:** State Key Laboratory of Optical Technologies on Nano-Fabrication and Micro-Engineering, Institute of Optics and Electronics, Chinese Academy of Sciences, China; School of Optoelectronics, University of Chinese Academy of Sciences, China

One of the ultimate goals of robots is the realization of AI human-behaved assistants. The emergence of intelligent indoor robotics is positioned to revolutionize the human experience. In recent times, such robots have demonstrated their utility in various sectors such as manufacturing, medical care, home healthcare, and others [1,2]. Despite these advancements, indoor robotics remain constrained in their capacity to offer a comprehensive field of view, accommodate varying dynamic ranges, operate in divergent environmental contexts, protect privacy, maintain acceptable cruise times, and support acceptable payloads [3,4]. Thus, the ability of robots to ‘see’ and ‘understand’ and to perceive and respond to complex indoor environments in a complex indoor context has the potential to assist humans in exploring the world.

**Figure 1.**
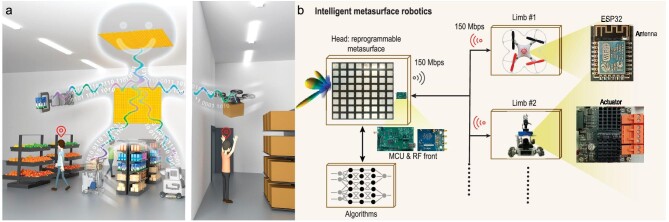
Conceptual illustration and system configuration of I2MR. (a) I2MR comprises two parts: brain and limbs; (b) system-level overview. Adapted with permission from Ref. [5].

In a recent article in the *National Science Review*, Zhao *et al*. proposed a novel approach to indoor robotics: intelligent indoor metasurface robotics (I2MR) [5]. This new approach comprises two main components that work together to create an efficient and effective robotic system. As shown in Fig. 1a, the first component of I2MR is the ‘brain’ of the robot, which has a metasurface-centered microwave perception modality and an AI-empowered data processor. The second component of I2MR is its ‘limbs,’ which include motorized vehicles or airborne drones powered by onboard batteries. This means that I2MR can ‘see’ around corners without creating images of the environment, thereby respecting privacy. The AI-powered data processor helps the robot to interpret the data obtained from its perception modality and make informed decisions about its next course of action. By combining metasurface technology, AI-powered data processing, and motorized limbs, I2MR has the potential to be a game-changer in terms of efficiency, privacy, and versatility.

Specifically, the programmable metasurface (Fig. 1b) of I2MR’s brain sequentially works in two ways. First, it generates configurational diversity to illuminate the indoor environment with pseudo-random patterns. The multipath propagation in a complex indoor environment boosts high localization precision on the order of millimeters through interferometric sensitivity. Based on the high-resolution 3D point clouds generated, the brain can interpret human posture, revealing health status or capturing action commands. Second, the brain focuses microwaves on the limb's wireless receiver and creates a high-capacity wireless link with the robotic limb. The brain consistently transmits tasks that are executed by the limbs.

This intelligent indoor robotics paradigm demonstrates the effective use of parameterized wireless environments to perform a range of sensing, localization, and communication tasks, all of which are coordinated by AI algorithms. The experimental results show that I2MR can control a computation-free robot in real-time in a smart way. The proposed method can be extended to other frequencies to develop more intelligent robots with more advanced functions. In the future, I2MR can be transferred to further important application areas of wireless network robotics entities, such as in factories. The results of the experiments provide a strong foundation for future developments in wireless network robotics, with potential applications in a variety of fields, such as manufacturing and healthcare.
